# *SOX2* Gene Regulates the Transcriptional Network of Oncogenes and Affects Tumorigenesis of Human Lung Cancer Cells

**DOI:** 10.1371/journal.pone.0036326

**Published:** 2012-05-15

**Authors:** Si Chen, Yingxi Xu, Yanan Chen, Xuefei Li, Wenjun Mou, Lina Wang, Yanhua Liu, Ralph A. Reisfeld, Rong Xiang, Dan Lv, Na Li

**Affiliations:** 1 School of Medicine, Nankai University, Tianjin, China; 2 Department of Immunology and Microbial Science, The Scripps Research Institute, La Jolla, California, United States of America; The Chinese University of Hong Kong, Hong Kong

## Abstract

Recent studies demonstrated that cancer stem cells (CSCs) have higher tumorigenesis properties than those of differentiated cancer cells and that transcriptional factor-*SOX2* plays a vital role in maintaining the unique properties of CSCs; however, the function and underlying mechanism of SOX2 in carcinogenesis of lung cancer are still elusive. This study applied immunohistochemistry to analyze the expression of SOX2 in human lung tissues of normal individuals as well as patients with adenocarcinoma, squamous cell carcinoma, and large cell and small cell carcinoma and demonstrated specific overexpression of SOX2 in all types of lung cancer tissues. This finding supports the notion that SOX2 contributes to the tumorigenesis of lung cancer cells and can be used as a diagnostic probe. In addition, obviously higher expression of oncogenes c-*MYC, WNT1, WNT2*, and *NOTCH1* was detected in side population (SP) cells than in non-side population (NSP) cells of human lung adenocarcinoma cell line-A549, revealing a possible mechanism for the tenacious tumorigenic potential of CSCs. To further elucidate the function of *SOX2* in tumorigenesis of cancer cells, A549 cells were established with expression of luciferase and doxycycline-inducible shRNA targeting *SOX2*. We found silencing of *SOX2* gene reduces the tumorigenic property of A549 cells with attenuated expression of c-MYC, WNT1, WNT2, and NOTCH1 in xenografted NOD/SCID mice. By using the RNA-Seq method, an additional 246 target cancer genes of SOX2 were revealed. These results present evidence that SOX2 may regulate the expression of oncogenes in CSCs to promote the development of human lung cancer.

## Introduction

Cancer stem cells (CSCs) represent a very small population of cancer cells from which tumors originates. They possess the same unique character as embryonic stem (ES) cells, such as clonogenicity, pluripotency and self-renewal and thus have the ability to initiate a tumor, sustain its growth and be responsible for cancer recurrence [1]. Recent studies have shown that CSCs like cell subpopulations could be isolated from various cultured tumor cell lines or tissues by using the Hoechst33342 dye efflux method to separate side population (SP) cells [2] or by sorting cells expressing specific stem cell surface markers, such as CD133(+), CD44(+), CD34(+) and CD38(+) [3]–[5] et al.

Lung cancer represents the most common cause of cancer-related lethality in both men and women throughout the world with very low five-year survival rates, even after clinical therapy [6], [7]. This malignancy is usually divided into different histological types according to the phenotypes of cells from which the tumor arises, including squamous cell carcinoma (SCC), adenocarcinoma and neuroendocrine carcinoma, such as small cell lung cancer (SCLC) as well as large cell lung cancer [8]. Adenocarcinoma, SCC and large cell lung cancer are also collectively named non-small cell lung cancer (NSCLC), representing the most common types of lung cancer with lower growth rate and spread speed than those of SCLC. Among NSCLC, peripheral adenocarcinoma is the leading subtype which accounts for approximately 80% of cases in lung cancer patients [9]. Several studies showed that CD133 (+), CD44 (+) and CD87 (+) can be used as surface markers to identify CSCs in lung cancer [10]–[12]. Recent studies reported isolated SP from both a mouse tumor model [13] and a variety of lung cancer cell lines by using the Hoechst dye efflux method [14]–[16]. It was found that isolated SP cells show higher expression levels of stem cell genes, such as *SOX2* and *OCT4* and tumorigenesis properties than NSP cells [2].

The important function of transcription factor *SOX2* in maintaining the unique properties of ES cells and CSCs has been extensively investigated. It was also established that induced pluripotent stem (iPS) or pluripotent cancer (iPC) cells could be generated by co-transfection of *Sox2* cDNA with other transcription factors such as *Oct4*, *Klf4* and *c-Myc* into fibroblast or cancer cells [17]–[20]. In fact, SOX2 was highly expressed in isolated CSCs like cells at both mRNA and protein levels. Extensive studies revealed that SOX2 regulates the complex transcriptional network to maintain the unique characteristics of stem cells [21] and the anti-apoptosis property of CSCs [15], [22]. Consequently, targeting of SOX2 is a promising strategy for tumor therapy. Although numerous investigations of clinically-derived tumor tissues reported the specific overexpression of SOX2 in certain types of tumor tissues, such as prostate and breast cancers [22], [23] and indicated its importance for tumorigenesis, the underlying mechanism for the tumorigenic property of *SOX2* gene is still largely unknown.

Oncogenes play important roles in the development of carcinoma. Among them, *NOTCH*, *WNT* and *c-MYC* are well-established oncogenes in the initiation and progression of lung cancer cells. It was reported that WNT family proteins-WNT1, WNT2 and NOTCH proteins -NOTCH1, NOTCH3 as well as their downstream protein HES-1 are overexpressed in NSCLC cell lines or tissues [24]–[31]. Overexpression of these oncogenes or activation of their signal pathways induced lung carcinoma [32], [33]. As such, targeting of these genes by using siRNA/shRNA, mutation, specific inhibitors or monoclonal antibodies could inhibit tumor growth and induce apoptosis in lung cancer cell lines in experimental mouse models [4], [34]–[36]. Aside from their important role in tumorigenesis of lung cancer, these oncogenes also formed a functional interaction network. It was also reported that NOTCH1 induces the expression of c-MYC, in addition, both proteins regulate the expression of same target genes participating in cell growth regulation [37]. c-MYC was also revealed to be the downstream target of WNT/β-Catenin signaling and further studies showed the promoter of *c-MYC* to align with WNT/β-Catenin responsive enhancers [38], revealing a possible regulatory mechanism of WNT signaling on c-MYC.

In view of the important contributions of SOX2 in maintaining the stemness property of CSCs, we hypothesize that SOX2 might govern the transcriptional network of oncogenes to affect the tumorigenesis of lung carcinoma. Our results thus far demonstrate that SOX2 regulates the transcriptional network of oncogenes, including *WNT1, WNT2, NOTCH1 and c-MYC* and promotes the tumorigenesis of human lung cancer cells. This study also supports the notions that targeting of SOX2 is an effective strategy for lung cancer therapy.

## Materials and Methods

### Ethics Statement

All animal experiments were performed strictly under the guidelines on laboratory animals of Nankai University and were approved by the Institute Research Ethics Committee at the Nankai University (Permit number: 10011). Mice were anesthetized with a mixture of oxygen/isoflurane before each experiment and all efforts were made to minimize their suffering. For human samples, the commercialized high-density tissue microarrays were purchased and the use of the human tissue in this study was approved by the Human Research Committee of Nankai University.

### Vector Construction

shRNA sequence for silencing human *SOX2* gene was searched and blasted using RNAi designer from the invitrogen website (https://rnaidesigner.invitrogen.com/rnaiexpress/index.jsp). One shRNA targeting human SOX2 was designed and chemically synthesized as shRNA-SOX2 (AAAAGGGACATGAT CAGCATGTATTGGATCCAATACATGCTGATCATGTCCC), and a scrambled sequence (AAAAGCTACACTATCGAGCAATTTTGGATCCAAAATTGCTCGATAGTGTAGC) was used as control for knockdown analysis. The palindromic DNA oligos were annealed to each other to form a double-strand oligo and ligated to the linearized pLV-H1TetO-GFP-Bsd (Cat# SORT-C03, Biosettia Inc., San Diego, CA) and pLV-H1-EF1α-puro (Cat# SORT-B19, Biosettia Inc., San Diego, CA) vector to generate circled pLV-H1TetO-shRNA-SOX2-GFP-Bsd and pLV-H1-EF1α-shRNA-SOX2-puro plasmid separately. The puromycin resistant cDNA from pLV-H1-EF1α-shRNA-SOX2-puro plasmid was cut by Nhe Ι and Sal Ι enzyme to replace the GFP-Bsd fragment in pLV-H1TetO-shRNA-SOX2-GFP-Bsd to generate the pLV-H1TetO-shRNA-SOX2-puro plasmid.

### Cell Culture

Wild types (Wt) of A549 and H460 cells were obtained from ATCC. A549-Wt cells were infected with lentivirus encoding the firefly luciferase (FL) gene (Cat. # GlowCell-14b-1, Biosettia, SanDiego, CA) and selected by 10 µg/ml Blasticidin (Bsd) to generate A549 cells with stable overexpression of FL (A549-FL). A549-Wt, H460-Wt and A549-FL cells were infected with lentivirus carrying pLV-H1TetO-shRNA-SOX2-puro plasmid (Biosettia, SanDiego, CA), followed by clonal selection using puromycin (10 µg/ml for A549 and 2 µg/ml for H460) to generate a polyclone of A549, H460 or A549-FL cells with stable expression of Dox inducible shRNA-SOX2 (A549-H1tetO-shRNA-SOX2, H460-H1tetO-shRNA-SOX2 or A549-FL/H1tetO-shRNA-SOX2). A549 and H460 cells were maintained in F12K and RPMI 1640 media supplemented with 10% fetal bovine serum in the presence of 100 u/ml penicillin and 0.1 mg/ml streptomycin separately. For control purposes, A549-Wt, H460-Wt and A549-FL were infected with lentivirus carrying scrambled shRNA and subjected to identical clone selection procedures to generate the stable control cell lines A549-H1tetO-shRNA-Con, H460-H1tetO-shRNA-Con and A549-FL/H1tetO-shRNA-Con.

**Table 1:** The primers used for RT-PCR and real-time RT-PCR.

WNT1	Forward primer 5′- CCCGGTTATTCGCCCACCCG -3′
	Backward primer 5′- CAAGGGGTCTCCCGCGGAGA -3′
WNT2	Forward primer 5′- ACAGCAGGCCGTGTGTGCAA -3′
	Backward primer 5′- AGGCAGTCCTGACAGCGCAC -3′
NOTCH1	Forward primer 5′- CGTCCGTGCCCCTCAACCAC -3′
	Backward primer 5′- CAGGACGGTGCTGGTGCCAG -3′
c-MYC	Forward primer 5′- CGCCCTCCTACGTTGCGGTC -3′
	Backward primer 5′- CGTCGTCCGGGTCGCAGATG -3′
SOX2	Forward primer 5′- AAAACAGCCCGGACCGCGTC -3′
	Backward primer 5′- CTCGTCGATGAACGGCCGCT -3′
NANOG	Forward primer 5′- ACCTCAGCCTCCAGCAGATGCA -3′
	Backward primer 5′- GGTGCTGAGGCCTTCTGCGT -3′
OCT4	Forward primer 5′- AAGCGATCAAGCAGCC -3′
	Backward primer 5′- GGAAAGGGACCGAGGAGTA -3′
ABCB1	Forward primer 5′- TTGAAGGGGACCGCAATGGAGGA -3′
	Backward primer 5′- GTCCAGCCCCATGGATGATGGC -3′
ABCG2	Forward primer 5′- CACCAATGGCTTCCCCGCGAC -3′
	Backward primer 5′- GGGTCCCAGGATGGCGTTGAGA -3′
β-actin	Forward primer 5′- GGCATCCACGAAACTACCTT -3′
	Backward primer 5′- CTCGTCATACTCCTGCTTGC -3′

### Immunohistochemistry and Tissue Microarrays

The expression of SOX2 in high-density tissue microarrays (Cat. # BC04119b, LC727, LC2085b, LC2161, Alenabio, Xi’an, China PR) was detected by the standard biotin-avidin-complex method with monoclonal mouse antibody against SOX2 (Cat. # ab75485, Abcam Inc, Cambridge, UK) at a 1∶200 dilution. The images were recorded by Olympus BX51 Epi-fluorescent microscopy under a 10× or 40× objective (Olympus Co. Tokyo, Japan).

**Table 2:** The correlation of SOX2 with clinical status of patient with lung cancer.

SOX2 (+) cell counts	≤10%	11–30%	31–49%	≥50%	*p* Value
**Tumorigenesis**					<0.001
Normal/Paracarcinoma	31	6	1	0	
Tumor	9	42	82	284	
* Adenocarcinoma*	4	25	44	127	
* SCC*	0	15	31	104	
* Large cell carcinoma*	3	1	5	22	
* SCLC*	2	1	2	31	
**Gender**					0.322
Male	32	35	66	201	
Female	8	13	17	83	
**Age at diagnosis**					0.024
<65	9	33	50	207	
≥65	0	9	32	77	
**Histological phenotype**					0.011
NSCLC	7	41	80	253	
SCLC	2	1	2	31	
**TNM stage**					
***NSCLC***					0.705
Ι-ΙΙ	7	34	60	182	
ΙΙΙ-ΙV	0	5	10	33	
***SCLC***					0.327
Ι-ΙΙ	1	0	1	20	
ΙΙΙ-ΙV	0	1	1	7	
**Histological grade**					
***Adenocarcinoma***					0.002
Grade 1	2	11	11	15	
Grade 2	1	6	16	54	
Grade 3	1	4	13	50	
**Localization of SOX2**					<0.001
Cytoplasma	40	19	26	53	
Nuclear + Cytoplasma	0	29	57	231	

### Flow Cytometry Analysis and Sorting

A549-Wt cells were harvested and resuspended in pre-warmed DMEM+ buffer (DMEM with 2% FBS and 10 mM HEPES buffer) at a density of 1.0×10^6^ cells/ml. Hoechst 33342 dye was added at a final concentration of 10 µg/ml in the presence or absence of 10 µM Fumitremorgin C (FTC). Cell samples were placed in a 37°C water bath for 60 minutes (min) and mixed every 10 min. Cells were collected and resuspended in cold HBSS+ buffer (Hanks’ Balanced Salt Solution with 2% FBS and 10 mM HEPES buffer). At the end of the staining period, cells were resuspended in cold HBSS+ buffer containing 2 µg/ml propidium iodide (PI) for dead cell discrimination. The Hoechst dye was excited with a UV laser at 355 nm, and its fluorescence measured with a 460/50 BP filter (Hoechst Blue) and a 670/30 BP filter (Hoechst Red).

**Table 3:** The most enriched 20 signaling pathways of SOX2 target genes in KEGG database.

#	Pathway	Count of DEG with pathway annotation	Pvalue
1	Ribosome	27	8.18E-09
2	Aminoacyl-tRNA biosynthesis	14	8.31E-06
3	Protein processing in endoplasmic reticulum	24	0.00067961
4	Renal cell carcinoma	15	0.00114849
5	Metabolic pathways	129	0.00132165
6	Bladder cancer	11	0.0027594
7	Insulin signaling pathway	23	0.0031735
8	Vibrio cholerae infection	12	0.00394879
9	Gap junction	16	0.00487479
10	Pathways in cancer	45	0.00773737
11	Lysosome	20	0.00824269
12	Glycosaminoglycan biosynthesis - keratan sulfate	5	0.00859688
13	Protein export	6	0.00925981
14	Vasopressin-regulated water reabsorption	9	0.01188259
15	Arginine and proline metabolism	10	0.01337271
16	DNA replication	8	0.01393426
17	Shigellosis	12	0.01882112
18	Phosphatidylinositol signaling system	13	0.01897797
19	Glycerolipid metabolism	8	0.0191644
20	Alanine, aspartate and glutamate metabolism	7	0.01921653

DEG: differentially expressed genes.

### RT-PCR and Real-time RT-PCR

Total mRNAs from A549 cells were isolated by TRIzol reagent (Cat. #15596-018, Invitrogen Inc, Carlsbad, CA) and reverse-transcribed into cDNAs with MMLV reverse transcriptase (Promega, Madison, MI). Following this, semi-quantitive RT-PCR was performed to detect the mRNA expression levels of *NOTCH1*, *WNT1*, *WNT2*, *c-MYC*, *SOX2*, *OCT4*, *NANOG*, *ABCB1* and *ABCG2*. For an equal loading control, mRNA of human *β-actin* was tested at the same time. Primers used for both experiments are summarized in **Table 1**. Real time RT-PCR was performed on Opticon (Bio-Rad, Hercules, CA) in 25 µl reaction volumes by using TransStart Green qPCR SuperMix Kit (TransGen Biotech, Beijing, China, PR). The 2^−ΔΔCt^ method was used to determine the relative mRNA folding changes. Statistical results were averaged from three independent experiments performed in triplicate.

**Figure 1:**
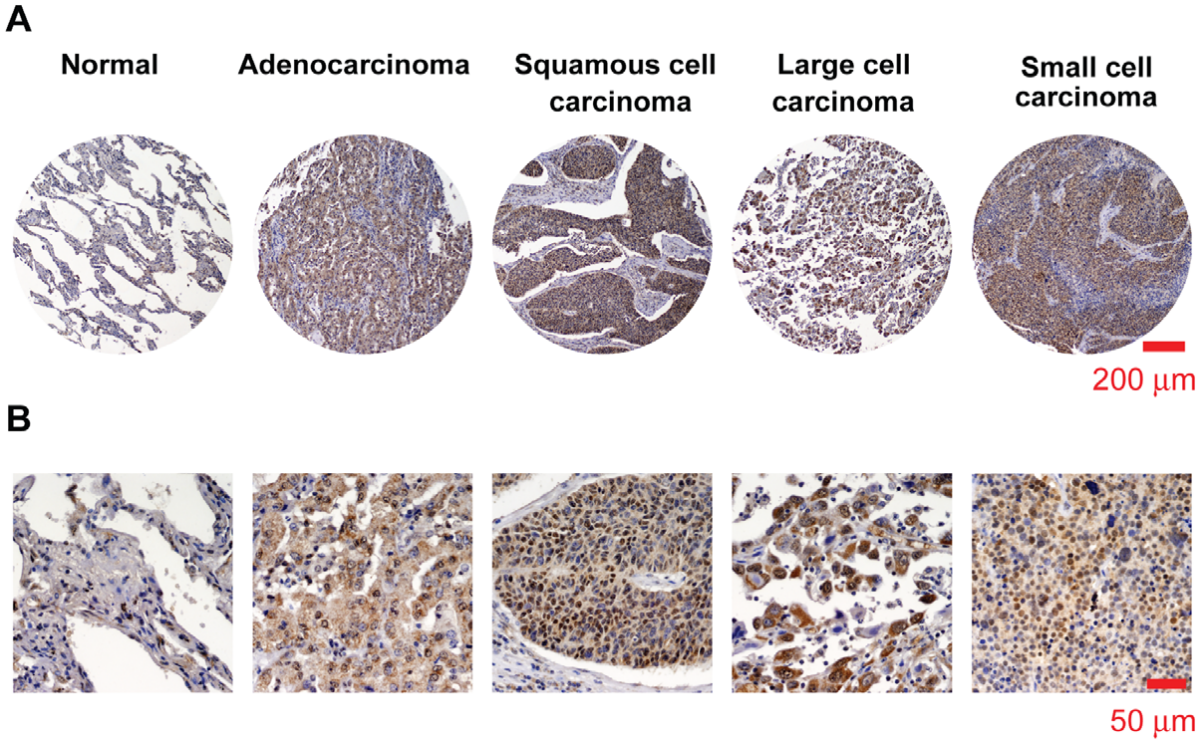
Immunohistochemical staining of SOX2 (brown) in human lung tissue of normal individuals, and patients with squamous lung carcinoma, adenocarcinoma lung cancer, small cell lung cancer and large cell lung cancer in paraffin-embedded human lung cancer tissue microarray. The representative staining of SOX2 in these tissues was subjected separately to microscopy images under 10× (**A**) and 40× objective (**B**).

### Western Blotting

Cell lysates from A549 and H460 cell lines were prepared with RIPA buffer in the presence of protease inhibitor cocktails as described previously [22]. Protein (20 µg) was loaded onto 5–12% Tris-Acrylamide gels and blotted with antibodies that included: polyclonal anti-SOX2, WNT2 (Cat.# sc-20088, sc-50361, Santa Cruz Biotechnology, Inc., Santa Cruz, CA), WNT1 (Cat.# ab85060, Abcam Inc, Cambridge, UK), monoclonal anti-NOTCH1 (Cat.# 3608, Cell Signal Technology Inc, Danvers, MA), c-MYC, β-actin (Cat.# sc-40, sc-47778, Santa Cruz Biotechnology, Inc. Santa Cruz, CA), and horseradish peroxidase-conjugated secondary antibodies. Blotting results were detected by an ECL chemiluminescence kit (Millipore, Billerica, MA).

**Figure 2:**
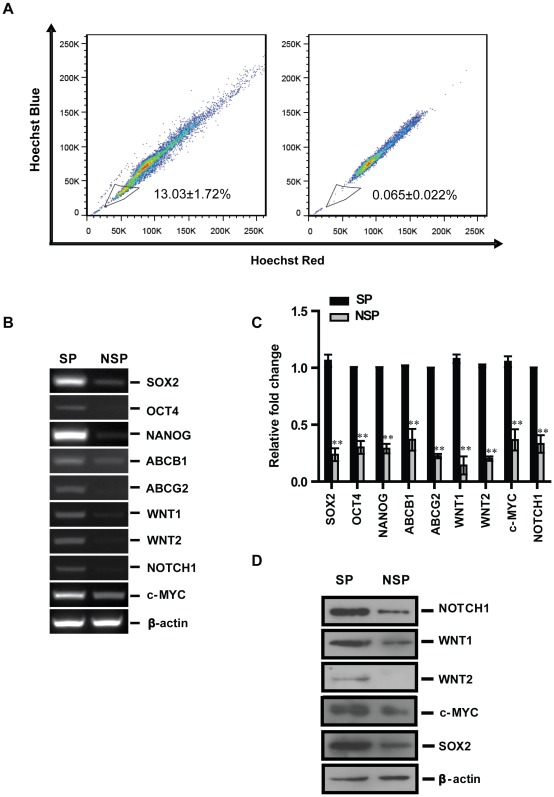
Oncogenes *WNT1*, *WNT2*, *c-MYC* and *NOTCH1* are specifically overexpressed in SP of human lung adenocarcinoma cell line A549. **A**. SP cells in A549 were detected and separated by FACS using the Hoechst33342 dye efflux method (left) and verified by their failure to efflux this dye after incubation with FTC, a specific inhibitor of multidrug transporter-ABCG2 (right). This figure represents 1 of 3 experiments. The mRNA expression levels of oncogenes and stem cell genes were compared between the SP and NSP cells by using semi-quantitive RT-PCR, real-time RT-PCR in **B** and **C** separately, n = 3. **D**. Western blotting results of WNT1, WNT2, NOTCH1, c-MYC and SOX2 proteins in SP and NSP of A549 cells.

### Tumor Xenograft

Male NOD/SCID mice at 6–8 weeks age were separated randomly into two groups (n = 5 for each group). 1×10^6^ A549-FL/H1tetO-shRNA-Con or A549-FL/H1tetO-shRNA-SOX2 cells were xenografted into each mouse through tail vein injection. All mice were fed with 0.2 mg/ml Dox plus 0.05% sucrose in the drinking water from the first day after inoculation.

**Figure 3:**
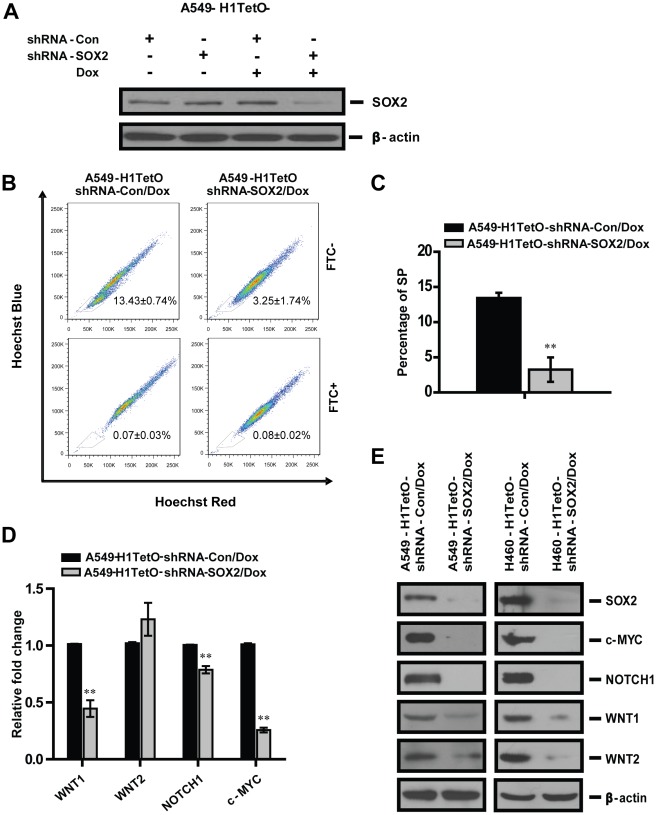
SOX2 regulates the expression of oncogenes in both A549 and H460 cells. **A**. A549-H1tetO-shRNA-SOX2 and control cells were incubated with Dox for 4 days and silencing of *SOX2* gene was confirmed by Western blotting. **B**. FACS analysis of the SP in A549 cells with or without *SOX2* silencing. The SP cells in A549 were confirmed by their ability to efflux Hoechst 33342 dye in the absence of FTC (up panel, FTC-), but fail to efflux the dye after incubation with FTC (low panel, FTC+). **C**. The percentage of SP in A549 cells from each experiment group was averaged from three independent experiments and plotted. **D**. Real-time RT-PCR was used to compare mRNA expression levels of oncogenes in A549 cell line with or without down-regulation of SOX2, n = 3. **E**. Protein expression levels of *WNT1*, *WNT2*, *NOTCH1* and *c-MYC* genes were detected in A549 and H460 cells with *SOX2* silencing by Western blotting.

### Bioluminescent Image

Mice were anesthetized with a mixture of oxygen/isoflurane and received luciferin (Cat. # 119222, Caliper Life Sciences, Hopkinton, MA) i.p at 150 µg/g body weight. Bioluminescent signals were measured 10 min later with an IVIS 100 imaging system and quantified with Living Image Software (Xenogen, Alameda, CA).

**Figure 4:**
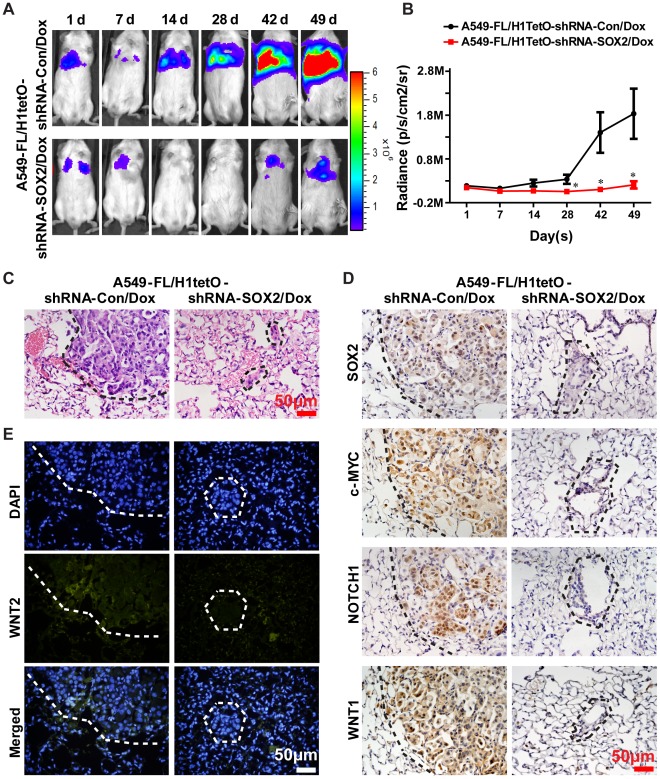
Silencing of *SOX2* inhibits tumorigenesis and regulates expression of oncogenes in vivo. **A**. A549 cells were injected via the tail vein into NOD/SCID mice and the bioluminescence images of xenografted tumor were taken at the times indicated. **B**. Bioluminescence intensity was measured and plotted, n = 5. **C**. HE staining was used to detect xenografted tumors in lung tissues of xenografted NOD/SCID mice. **D**. Immunohistochemistry staining of SOX2, c-MYC, NOTCH1 and WNT1 (all shown in brown color) from lung tissues of xenografted NOD/SCID mice in situ. **E.** Immunofluorescence of WNT2 (green) in xenografted murine lung tissues. All microscopy images were recorded under a 40× objective. The figures in **C**, **D** and **E** represent 1 of 5 experiments. The tumor regions in **C**, **D** and **E** were all circulated by dash lines.

### RNA-Seq

The A549-H1tetO-shRNA-SOX2 and its control cells were collected after being incubated with Dox for 4 days. 4 µg total RNA from each sample was extracted by TRIzol reagent. Oligo(dT) magnetic beads adsorption method was used to purify mRNA, their transcriptome data were profiled and compared following standard protocols (digital gene expression, DGE, 3 million reads, Beijing Genomics Institute at Shenzhen, China). The cancer genes were selected from 7 databases summarized in the website: http://microb230.med.upenn.edu/protocols/cancergenes.html and those genes with false discovery rate (FDR) <0.001 were selected out as the target genes of SOX2. RNA-Seq data have been deposited to NCBI Gene Expression Omnibus (GEO) database (GSE36597; reviewer access link: http://www.ncbi.nlm.nih.gov/geo/query/acc.cgi?token=druvhickewcwcvy&acc=GSE36597).

**Figure 5:**
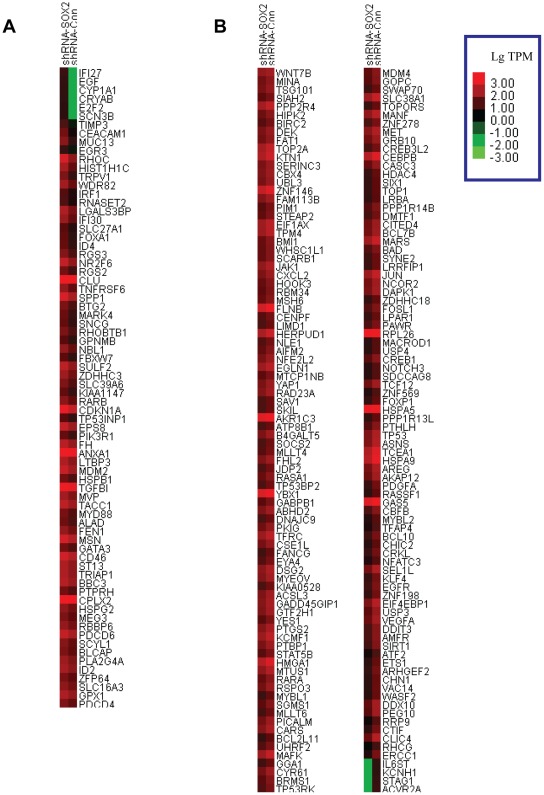
Cluster image of the other target cancer genes of SOX2. Standardized TPM (transcripts per million clean tags) values were applied to compare the other target cancer genes expression levels between A549 cells with *SOX2* silencing (shRNA-SOX2) and their control (shRNA-Con). The transcription of each gene is represented by a square with a color that codes for the values of Lg TPM. Specifically, bright green represents low expression, bright red represents strong expression. The target genes whose transcriptions are up-regulated with the silencing of *SOX2* were presented in **A** and those down-regulated genes were presented in **B**.

### Statistical Analysis

Values were expressed as Means ± S.E.M. Significance was determined by χ^2^ test in **Table 2** and hypergeometric test in **Table 3**, others were determined by Student’s t-test. A value of *p*<0.05 was used as the criterion for statistical significance. * indicates significant difference with *p*<0.05, ** indicates significant difference with *p*<0.01.

## Results

### SOX2 is Specifically Expressed in Human Lung Cancer Tissues

To determine whether SOX2 contributes to tumorigenesis of lung cancer, an immunohistochemical method was used to detect expression of SOX2 in human lung tissues of normal/paracarcinoma (n = 38), adenocarcinoma (n = 200), SCC (n = 150), SCLC (n = 36) and large cell lung cancer (n = 31). The correlation of SOX2 with clinical status of patients with lung cancer was summarized in **Table 2**. Predominantly higher expression of SOX2 was found in both NSCLC and SCLC than in normal/paracarcinoma tissues (*p*<0.001), indicating the important role of SOX2 in the tumorigenesis of lung cancer. Statistically results significant showed the number of SOX2 positive cells to be positively correlated with age at diagnosis (*p* = 0.024). Moreover, SCLC tissues revealed a higher expression level of SOX2 than NSCLC tissues (*p* = 0.011). There was a positive correlation between SOX2 and the pathological degree of human adenocarcinoma (*p* = 0.002), indicating that SOX2 may inhibit the differentiation of adenocarcinoma cells. From results of immunohistochemical staining (**Fig. 1**), we found either cytosol or both nuclei and cytosol localization of SOX2 in cancer cells, which was consistent with our previous findings in human prostate cancer cells [22]. In addition, most of the tumor tissues with high expression level of SOX2 showed both nuclear and cytosol localization of SOX2; in contrast, those tissue with lower expression level of SOX2 always revealed only cytosol localization. The correlation between SOX2 expression level and its localization was significant (*p*<0.001); however, there was no statistically significant relationship between SOX2 positive cancer cell counts and TNM stages or gender.

### Oncogenes WNT1, WNT2, NOTCH1 and c-MYC are Overexpressed at Both mRNA and Protein Levels in SP Cells of the A549 Cell Line

Following identification of specific overexpression of SOX2 in human lung tumor tissues, the expression of SOX2 and well-established oncogenes were compared between SP and none side population (NSP) in A549 cells. SP cells are CSCs-like cells that show higher tumorigenesis and chemo-resistant properties than those of NSP cells [39], [40]. Since they express high expression levels of ATP-binding cassette (ABC) transporter family members, which help them to extrude the Hoechst 33342 dye and some drugs more effectively, SP cells could be isolated by the Hoechst 33342 efflux method using FACS. As shown in **Fig. 2A** **,** approximately, 13% of cells could be isolated as SP by the Hoechst dye efflux method. Stemness of isolated SP cells was proven by their specific overexpression of *SOX2*, *OCT4, NANOG, ABCB1* and *ABCG2* genes. Significantly higher expression levels of *WNT1*, *WNT2*, *NOTCH1* and *c-MYC* were detected in SP cells than those observed in NSP cells at both mRNA (**Fig. 2B** **,** **Fig. 2C**) and protein levels (**Fig. 2D**), suggesting the molecular mechanism underlying the high tumorigenic property of CSCs.

### SOX2 Regulates the Expression of Oncogenes in A549 and H460 Cells

Since *SOX2* is an important transcriptional factor that maintains the function of CSCs, we hypothesized that SOX2 might regulate the transcription of certain oncogenes in tumor cells. To test this contention, A549 cells were established with stable expression of doxycycline (Dox) inducible shRNA targeting SOX2 (A549-H1tetO-shRNA-SOX2). The inducible knockdown of SOX2 was confirmed by Western blotting after 4 days’ incubation of A549 cells with Dox, and 70% silencing efficiency of shRNA was demonstrated in the expression of *SOX2* gene (**Fig. 3A**). At the same time, significant reduced percentage of SP in A549 cells with *SOX2* gene silencing was revealed (**Fig. 3B and 3C**), demonstrating the important function of SOX2 in maintaining the CSC properties of A549 cells. In addition, this finding also supported the contribution of SOX2 to the tumorigenesis capacity of lung cancer cells since it was well established that the SP has higher tumorigenesis property than NSP in various types of lung cancer cells [16]. By using real time RT-PCR and Western blotting, significant reduced expressions of *WNT1*, *NOTCH1* and *c-MYC* at both mRNA and protein levels were shown in A549 cells with SOX2 down-regulation (**Fig. 3D and 3E**). An interesting phenomenon observed was that although mRNA expression of *WNT2* was slightly elevated upon silencing of *SOX2*, its protein expression level decreased, indicating a translational inhibition of *WNT2* in A549 cells with *SOX2* silencing. The same regulatory effects of SOX2 on the expression of *WNT1*, *WNT2, NOTCH1* and *c-MYC* were also observed in the human large cell lung carcinoma cell line H460 with *SOX2* silencing (**Fig. 3E**).This finding indicates a universal regulatory mechanism of SOX2 on the oncogene network of both human lung cell lines. These findings support the notion that SOX2 may regulate the transcription of key oncogenes in lung cancer to promote tumorigenesis.

### Silencing of SOX2 Attenuates Tumorigenesis of Human Lung Cancer Cells in Xenografted NOD/SCID Mice

Following the identification of SOX2’s function on tumorigenesis and its regulatory effect on expression of oncogenes in human lung cancer cells, we next explored its function in vivo. For this purpose, A549-FL/H1tetO-shRNA-SOX2 and control cells were inoculated into the NOD/SCID mice by tail vein injection. The presence of A549-FL cells was monitored by noninvasive bioluminescence imaging. The mice xenografted with A549-FL/H1tetO-shRNA-SOX2 and A549-FL/H1tetO-shRNA-Con cells were fed with Dox through drinking water from the first day after inoculation to induce expression of shRNA. As observed in **Fig. 4A and 4B**, same amount of bioluminescence signals could be detected in mice 1 day after injection, indicating that the same number of lung tumor cells became trapped in lung capillaries in both experimental groups. Subsequent decrease in bioluminescence signals was detected in the 7 following days due to a failing in survival of xenografted lung cancer cells [41]. Progressively increasing signals could be observed in mice xenografted with A549-FL/H1tetO-shRNA-Con cells after 14 days of treatment, indicating that these cells had succeeded in homing and proliferating. Twenty-one days after tumor cell challenge, 5 of 5 A549-FL/H1tetO-shRNA-Con xenografted mice developed tumor, but only 2 of 5 A549-FL/H1tetO-shRNA-SOX2 xenografted mice developed tumor 42 days after initial tumor cell challenge. Xenografted lung tumors in NOD/SCID mice were confirmed by hematoxylin and eosin (HE) stainings as shown in **Fig. 4C**. The protein expression levels of *SOX2* and oncogenes in murine lung tissues were assayed by immunohistochemistry or immunofluorescence methods. We found a reduced tumorigenesis potential of A549 cells in vivo as well as a significantly attenuated expression of WNT1, WNT2, NOTCH1 and c-MYC after silencing of the *SOX2* gene (**Fig. 4D** and **Fig. 4E** **).** Taken together, these results supported the tumorigenic property of SOX2 and its regulatory effect on oncogenes in vivo.

### SOX2 Regulates the Transcriptional Network of Oncogenes

Having identified the function of SOX2 in the tumorigenesis property of lung cancer cells and its target oncogenes, we then tested all the other targeted genes by RNA-Seq and found 246 cancer genes. Among them, 74 genes (30%) were up-regulated (**Fig. 5A**) and 172 genes (70%) were down-regulated (**Fig. 5B**) in A549 cells with *SOX2* silencing. We noticed the transcription levels of *NOTCH3* and *WNT 7B* are significantly reduced, revealing the comprehensive effects of SOX2 on the transcription of *WNT* and *NOTCH* gene families. In addition, the mRNA expression levels of well-established oncogenes such as *KLF4*, *EGFR*, *BCL10*, *JUN*, *YAP1* and *JAK1* et al. were significantly reduced when compared with control cells, but the transcriptions of *EGF*, *RHOC* et al. were greatly increased, demonstrating the complicated molecular mechanism in the tumorigenesis process of SOX2. In addition, our RNA-Seq result revealed 840 differentially expressed genes (DEG) with pathway annotation after *SOX2* silencing in A549 cells. Through enrichment analysis of SOX2 target genes in the cell signaling pathway database of Kyoto Encyclopedia of Genes and Genomes (KEGG), we found the pathway in cancer is one of the most significantly enriched ones (**Table 3** and **Fig. S1**). This result further supports the important function of SOX2 in development of tumor.

## Discussion

In this study, we used immunohistochemistry to systemically analyze the expression of SOX2 in various types of lung cancers and found that SOX2 is predominantly overexpressed in adenocarcinoma, SCC, large cell carcinomas and SCLC tissues, indicating that SOX2 can be used as a universal marker for the diagnosis of human lung cancer. Here we also present evidence indicating that SOX2 plays an important role in carcinogenesis of lung cancer.

Since the concept of CSC has been established, many studies supported this concept by demonstrating that CSCs or CSCs like cells are highly tumorigenic [16]; however, few studies have been performed to investigate the molecular mechanism of this phenomenon. Here we demonstrated a higher expression of *SOX2* and oncogenes- *NOTCH1, WNT1*, *WNT2* as well as *c-MYC* in SP cells than those in NSP cells, thus supporting a possible contribution of these oncogenes to the stemness property of CSCs.

Recent studies have revealed the complicated interaction between SOX2 and WNT signaling pathway. For example, it was reported that SOX2 antagonizes the WNT signaling to inhibit the differentiation of adult stem cells (ASCs) [42] and osteoblast lineage [43] and enhance the tumorigenesis and self renewal property of osteosarcomas [44] by promoting the transcription of negative regulator of WNT signaling, such as DKK1, APC and GSK3β. SOX2 was also reported to synergistically interacted with β-catenin, the downstream molecule of the WNT signal pathway, to regulate the transcription of a target gene to promote cell proliferation and tumorigenesis of human breast cancer [23]. However, as an important stem cell gene, the underlying molecular mechanism of SOX2’s function in tumorigenesis is comprehensive and still needs to be intensively investigated. In this regard, our results demonstrated that silencing of *SOX2* significantly reduces the protein expression level of oncogenes-*WNT1*, *WNT2*, *c-MYC* and *NOTCH1* in human lung cancer and further revealed other target cancer genes of SOX2. It is thus possible that SOX2 can cooperate with these important oncogenes to promote tumor occurrence. Recent study showed down-regulation of *SOX2* gene inhibits proliferation and induces apoptosis in tumor cells [15], [45], [46], we observed the same phenomenon in human lung cancer cell lines (data not shown), those may also be important factors that finally lead to the suppressed tumor growth in A549 cells; however, this study focused on the transcriptional changes of oncogenes and did not address other mechanisms.

Bioluminescence imaging is a novel diagnostic system that provides an easy and noninvasive visualization of the size and location of tumor cells in xenografted animals. Two types of models are usually applied to explore tumorigenicity of lung cancer cells: one is the intravenous metastasis model and the other is the subcutaneous flank tumor model [47], [48]. The first model was used in our study to observe SOX2’s function in the tumorigenesis of lung cancer in vivo, which reflected the whole process of homing and proliferation of tumor cells in lung. It was interesting to find that after 42 days of tumor cell inoculation, lung tumors could be detected in 2 of the 5 A549-FL/H1tetO-shRNA-SOX2 xenografted mice. From the immunohistochemistry stain of tumor tissues, the possibility that the tumor formed from “escaper” cells that not responsive to shRNA-SOX2 could be excluded, since the stain intensity of SOX2 was obviously decreased in the shRNA-SOX2 group. It is thus possible that the transcription of other oncogenes may not be affected or could even be unregulated by SOX2 and this may be the key reason leading to tumor occurrence in NOD/SCID mice. This possibility was verified by RNA-Seq screening of the targeted cancer genes of SOX2 by showing the up-regulated transcription of oncogenes after *SOX2* gene silencing. Discovery of these genes’ function in the occurrence of tumor after SOX2 down-regulation and use of combined therapies targeting them and the *SOX2* gene may improve the outcome of lung tumor therapy.

In summary, this study broadens our understanding of the molecular mechanism of tumorigenesis of both CSCs and SOX2. Our study supports the notion that silencing of the *SOX2* gene is an effective strategy for human lung cancer therapy.

## Supporting Information

Figure S1 **Pathway in cancer, one of the most significantly enriched pathways of SOX2 targets from KEGG cell signaling pathway database.** The frame showed the SOX2 target that was down-regulated (green) or up-regulated (red) upon *SOX2* silencing.
(DOC)
