# Supplementary figures and images for: SOX2 Gene Regulates the Transcriptional Network of Oncogenes and Affects Tumorigenesis of Human Lung Cancer Cells
Source: PLoS One. 2012 May 15;7(5):e36326. doi: 10.1371/journal.pone.0036326 (PMC3352903; doi:10.1371/journal.pone.0036326)

**Supplementary Data**

**Figure S1**


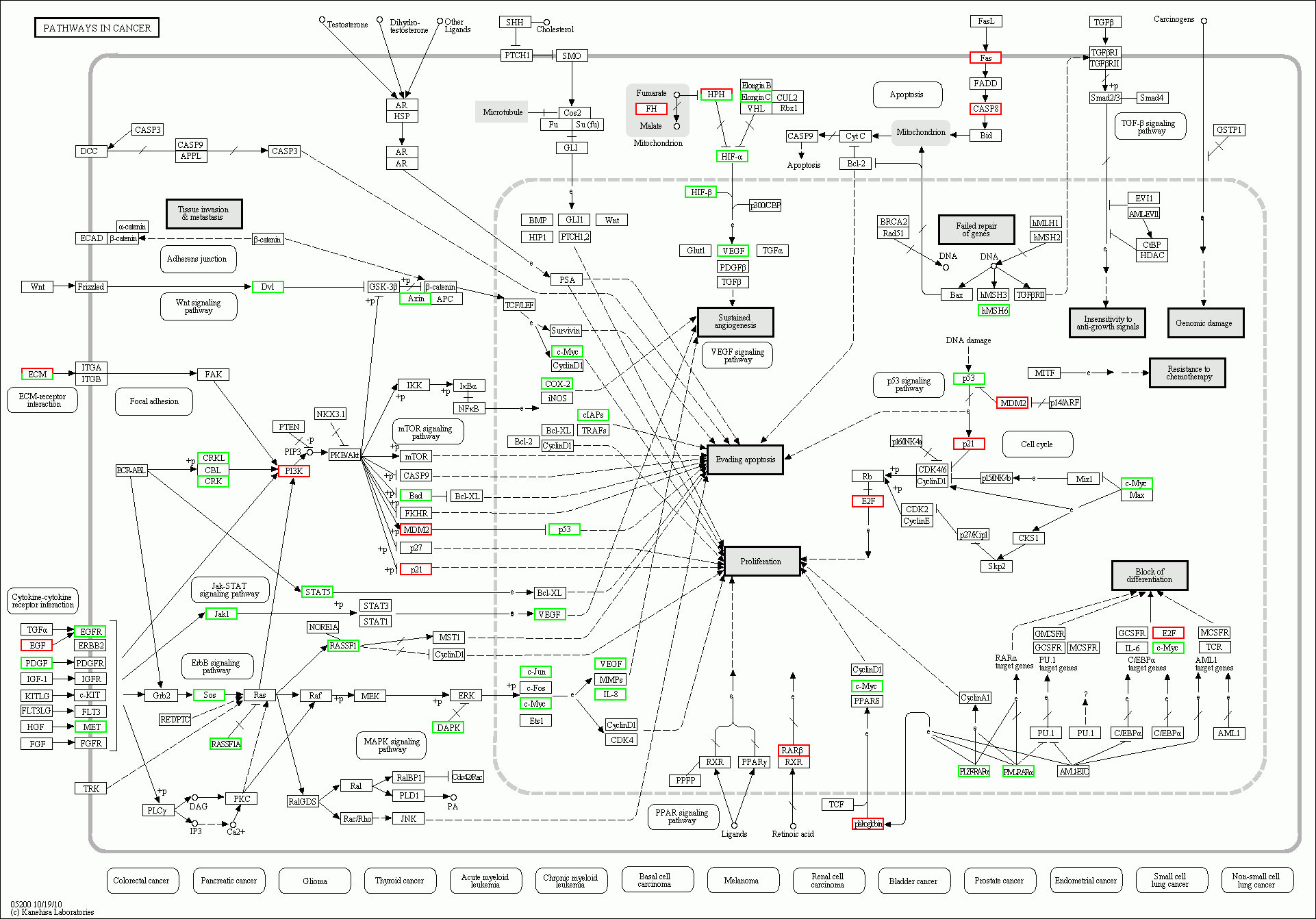

Supplement: Figure S1 — Pathway in cancer, one of the most significantly enriched pathways of SOX2 targets from KEGG cell signaling pathway database. The frame showed the SOX2 target that was down-regulated (green) or up-regulated (red) upon SOX2 silencing. (DOC) [file pone.0036326.s001.doc]
